# Isolation and Culture of Primary Human Dental Pulp Cells—A Description of Technical and Methodological Steps to Maximise Predictability and Yield

**DOI:** 10.3390/mps7020022

**Published:** 2024-03-01

**Authors:** Michaela Kearney, David E. McReynolds, Henry F. Duncan

**Affiliations:** Division of Restorative Dentistry & Periodontology, Dublin Dental University Hospital, Trinity College Dublin, University of Dublin, D02 PN40 Dublin, Ireland

**Keywords:** DPC isolation, dental pulp, regenerative endodontics, explant outgrowth, tissue culture, dental pulp stem cells

## Abstract

The dental pulp has critical functions in tooth development as well as an ongoing role in promoting and maintaining the vitality of teeth. In particular, its regenerative ability allows dental tissues to be restored following damage caused by traumatic injury or caries. Regenerative endodontic procedures aim to utilise these processes to stimulate dental pulp repair in a minimally invasive manner and reduce the need for more invasive procedures such as root canal treatment. Dental pulp is a source of dental pulp cells (DPCs), which has a subpopulation of dental pulp stem cells (DPSCs), which are attractive for use in regenerative medicine due to their high proliferation rate, ability to differentiate into multiple cell types, and their preserved vitality following cryopreservation. The development of next-generation clinical therapeutics that maximise the potential of dental pulp relies on strong empirical evidence arising from in vitro experimentation. Here, we describe a modified method for the efficient isolation of primary human DPCs from sound third molar teeth for culture using an explant outgrowth method on basement membrane-coated flasks, as well as using high-resolution macro-photography to illustrate the methods. Critically, steps are taken to minimise potential physical and mechanical trauma to the cells and maximise yield. Human DPCs cultured using this method can be further expanded in cell culture flasks to facilitate their use in various in vitro experimental procedures.

## 1. Introduction

The dental pulp is a connective tissue that occupies the centre of the tooth and has a wide range of functions related to maintaining the vitality and integrity of the tooth. In particular, the dental pulp plays a critical role in repair and regeneration by producing tertiary reactionary or reparative dentine in response to damage, such as physical trauma or a carious attack. These processes are mostly mediated by dental pulp stem cells (DPSCs), a subpopulation of mesenchymal stem cells (MSCs) that reside in the pulp, constituting approximately 1–5% of the adult dental pulp cell (DPC) population [1], and can differentiate into odontoblast-like cells in response to trauma, which then deposit tertiary dentine [2]. In addition to their ability to differentiate into odontoblast-like cells, DPSCs have also demonstrated the capacity to differentiate into a number of other cell types, including chondrocytes [3,4], adipocytes [5] and neurons [6]. For this reason, DPSCs are attractive for use in regenerative medicine, as evidenced by studies investigating their therapeutic potential in spinal cord injury [7], muscular dystrophy [8], as well as dental pulp repair [9]. The further development of therapeutics using DPSCs is heavily reliant on thorough in vitro experimentation, which itself is dependent on an effective protocol for the isolation and culture of human dental pulp cells (hDPCs).

There is currently a lack of consensus surrounding the optimal method for processing dental pulp tissue for the purpose of isolating DPCs, and later DPSCs. The use of a high-speed air-turbine dental drill to expose the pulp chamber, as is the protocol in a clinical setting, is commonly suggested [10,11]; however, it is likely that the considerable mechanical trauma could cause stress-induced genetic or physiological changes in the cells that could ultimately affect cell viability and downstream results. In order to reduce this risk, the method described here allows for exposure of the pulp chamber without direct contact with the pulp tissue. Following pulp extraction, either the explant outgrowth method or the enzymatic digestion method can be used for initial culture. The explant outgrowth method involves the outward growth of cells from a piece of pulp tissue deposited in a culture flask, while the enzymatic digestion method involves digesting minced tissue with an enzyme such as trypsin to obtain a single cell suspension, which can then be seeded in a basement-membrane-coated culture flask for further expansion. The optimal method for DPC isolation and culture should ideally minimise damage to the cells and maintain their in vivo characteristics. In this regard, it has been suggested that enzymatic digestion may result in a compromised cell membrane or loss of stem cell characteristics [12,13,14,15]; however, cells isolated by enzymatic digestion have been demonstrated to have higher proliferation rates than those isolated by a tissue outgrowth method [16]. In addition, dental pulp that has been processed via enzymatic digestion has been suggested to have a higher population of stem cells than pulp tissue processed using an explant outgrowth method, in a study which demonstrated the former to possess increased mineralisation potential [17]. As part of this study, both methods were used and compared with each other, and it was observed that the enzymatic digestion method did not consistently produce sufficient cell numbers for further expansion, as was the case when using the explant outgrowth method. 

In the current study, Matrigel^®^ is used as a basement membrane extract to promote explant attachment and initial cellular outgrowth. Matrigel^®^ is an extract of the Engelbreth-Holm-Swarm (EHS) mouse sarcoma, and is rich in laminin, growth factors, collagen IV, and other extracellular components. Due to its similarity to the extracellular environment found in many tissues, it is widely used as a basement membrane in cell culture to promote cell differentiation and proliferation. As part of the current study, dental pulp explants were initially transplanted into uncoated flasks; however, explant attachment and subsequent cell outgrowth was unpredictable and inefficient. Matrigel^®^ was subsequently chosen as a substrate due to its ability to promote cell proliferation. The use of collagen I was also considered, as it is the most prominent collagen form found in dental pulp and would therefore closely mimic the in vivo environment in which DPCs are located. However, Matrigel^®^ was ultimately chosen due to its ability to maintain stemness [18], which may be an attractive quality where DPSCs are to eventually be isolated from the DPC population. 

The primary aim of this methodological study is to provide a detailed description and visualisation of the methods required to extract dental pulp from the pulp chamber for culture using an explant outgrowth method. Critically, the pulp itself is not in direct contact with any surgical instrument, thereby reducing the risk of physical or mechanical trauma. Furthermore, a basement membrane matrix is used to improve explant attachment and encourage cell growth. Methods for subculturing cells to allow for expansion of hDPCs in culture are also described.

## 2. Experimental Design

**Figure 1:**
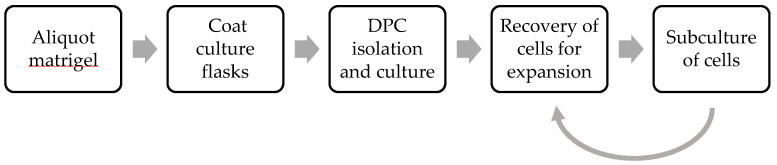
Methodological flowchart.

The protocol outlined here describes the isolation and culture of primary DPCs from human third molars (Figure 1). Third molars from which dental pulp is isolated should be free from caries or restorations, as these factors could alter the DPC phenotype. While samples can be obtained from consenting donors of any age, it should be noted that the pulp chamber decreases in size with increasing age, and so samples obtained from younger donors are likely to have a larger amount of pulp tissue present. Tissue culture flasks are initially coated with Corning^®^ Matrigel^®^ Basement Membrane Matrix prior to explant deposition, in order to aid attachment and cell growth. Pulp tissue is then extracted from sound third molars, using a method which has been optimised to avoid physical or mechanical trauma to the pulp tissue. Pulp tissue explants are subsequently deposited in Matrigel^®^-coated cell culture flasks for cell outgrowth. Methods to depolymerise the Matrigel^®^ matrix and recover the cells for further expansion are also provided. The steps are outlined in clear and concise detail, and high-resolution images are included to provide a visual aid.

### 2.1. Materials

1. Corning^®^ Matrigel^®^ Basement Membrane Matrix, LDEV-Free (Corning, New York, NY, USA; Cat. No.: 356234).
2. Dulbecco’s Modified Eagle Medium (DMEM), containing 2 mM (0.584 g/L) of L-Glutamine (Biowest, Nuaillé, France; Cat. No.: L0104) and supplemented with 1% Penicillin/Streptomycin (100 units/mL of penicillin with 100 μg/mL streptomycin) (Sigma-Aldrich, Arklow, Ireland; Cat. No.: P4333), 2.5 μg/mL Amphotericin B (Sigma-Aldrich, Arklow, Ireland; Cat. No.: A9528).
3. Sound third molars, extracted no more than 2 h prior to use.
4. Phosphate-buffered saline (PBS), pH 7.5: To be prepared by dissolving PBS tablet (Thermo Fisher Scientific, Dublin, Ireland; Cat. No.: BR0014G) in 500 mL ultrapure water. Autoclave the solution at 121 °C for 15 min and store at room temperature for no longer than 12 months.
5. PerioKin mouthwash (0.2% chlorhexidine) (Laboratorios KIN, Barcelona, Spain; Cat. No.: 7153).
6. Cell culture medium: DMEM supplemented with 20% (*v*/*v*) Foetal Calf Serum (FCS) (Biosera, Labtech International, East Sussex, UK; Cat. No.: FB-1001). Required volumes to be incubated at 37 °C before use in cell culture by placing in a water bath.
7. Trypsin-EDTA: 0.25% (*w*/*v*) Trypsin and 1 mM EDTA.4Na (Sigma-Aldrich, Arklow, Ireland; Cat. No.: T4049). Required volumes to be incubated at 37 °C in a water bath prior to use.
8. Corning^®^ Cell Recovery Solution (Corning, New York, NY, USA; Cat. No.: 354253).

### 2.2. Equipment

1. Ice bucket.
2. Pipette tips: 200 µL, 1000 µL (StarLab, Milton Keynes, UK; Cat. No.: S1113-1810, S1111-6811).
3. Sterile serological pipettes: 5 mL, 10 mL (Grenier Bio-One, Stonehouse, UK; Cat. No.: 606180, 607180).
4. Eppendorf tubes: 0.5 mL (Eppendorf, Hamburg, Germany; Cat. No.: 0030121023).
5. Sterile T25 flasks (Sarstedt, Leicester, UK; Cat. No.: 83.3910.302).
6. Centrifuge tubes: 50 mL (Abdos Labtech, New Delhi, India; Cat. No.: P10416)
7. Water bath (Sub Aqua 12 Plus, Grant Instruments, Royston, UK; Cat. No.: SBB Aqua 12 Plus).
8. Water-cooled diamond blade saw (IsoMet Low Speed Saw, Buehler, IL, USA; Cat. No.: 11-1280-170).
9. Sterile surgical gauze (Henry Schein, Dublin, Ireland; Cat. No.: 1201516).
10. Sterile surgical chisel (Henry Schein, Dublin, Ireland; Cat. No.: 1113696).
11. Sterile surgical mallet (Henry Schein, Dublin, Ireland; Cat. No.: 1120746).
12. Scalpel (Swann-Morton, Sheffield, UK; Cat. No.: 0503).
13. Sterile glass slides (Thermo Fisher Scientific, Dublin, Ireland; Cat. No.: 12373118).
14. Centrifuge (Universal 320, Hettich, Tuttlingen, Germany; Cat. No.: 1401).
15. Sterile T75 flasks (Sarstedt, Leicester, UK; Cat. No.: 83.3911.302).
16. Phase contrast microscope (Zeiss Axiovert 25, Oberkochen, Germany; Cat. No.: 491205 9804)
17. Standard 37 °C, 5% CO_2_ humidified tissue culture incubator (Sanyo, Osaka, Japan; Cat. No.; MCO-18AC(UV)).

## 3. Procedure

All solutions should be sterile. All equipment should be certified as nuclease-free by the supplier. All procedures should be carried out under aseptic conditions in a biological safety cabinet (Class II or higher).

### 3.1. Aliquoting Matrigel^®^


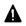
 **CRITICAL STEP** One day prior to aliquoting, store pipette tips, Eppendorf tubes and a tube rack at −80 °C overnight. When handling Matrigel^®^, all reagents and consumables must be kept on ice. Matrigel^®^ will solidify and adhere to any equipment above 10 °C. Change pipette tips regularly.

9. To thaw Matrigel^®^, submerge an unopened vial in ice in ice bucket. Close and seal ice bucket and store at 4 °C overnight.
10. Determine the protein concentration of the Matrigel^®^ solution as indicated on the specification sheet.

Note: The protein concentration of Matrigel^®^ varies lot-to-lot (in general ranging from 8 to 11 mg/mL). The protein concentration is indicated on the bottle or on the specification sheet. Confirm that the lot number on the bottle matches the lot number on the specification sheet before noting the protein concentration.

**Figure 2:**
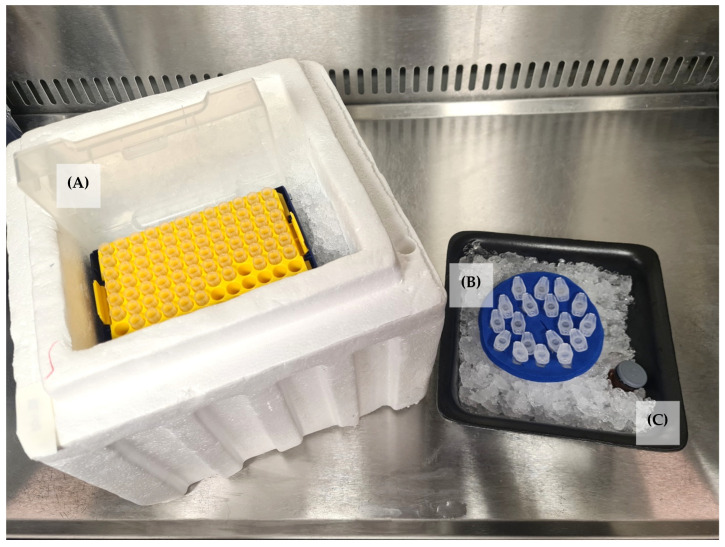
Setup required for handling and aliquoting of Matrigel^®^. All reagents and consumables are kept on ice. (**A**) Chilled pipette tips. (**B**) Sterile chilled 0.5ml Eppendorf tubes. (**C**) Thawed Matrigel^®^.

11. Calculate the volume of Matrigel^®^ corresponding to a 2 mg protein. For example, assuming a solution of Matrigel^®^ with a protein concentration of 8.9 mg/mL, the following applies:
  $$1\;mL\left(\frac{2\;mg}{8.9\;mg}\right)=0.2247\;mL$$
  Therefore, a 224.7 µL aliquot contains 2 mg protein.

12. Place chilled Eppendorf tubes in a chilled tube rack on ice. Place chilled pipette tips on ice (Figure 2).
13. Swirl Matrigel^®^ to ensure even dispersal.
14. Using chilled pipette tips, dispense the appropriate volume of Matrigel^®^ corresponding to 2 mg into chilled Eppendorf tubes.

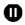
 **PAUSE STEP** Aliquots can be stored at −20 °C or −70 °C.

### 3.2. Coating Culture Flasks

1. Thaw Matrigel^®^ aliquots on ice.
2. Using a sterile cold pipette tip, dilute a 2 mg aliquot of Matrigel^®^ to 1 ml with chilled DMEM for a final concentration of 2 mg/mL (See Note 1, Appendix A).
3. Add the Matrigel^®^ solution to a sterile T25 flask and shake gently to ensure even coating.
4. Incubate at room temperature in a Class II biosafety cabinet overnight with the lid placed loosely on the flask to allow Matrigel^®^ coating to set.
5. Sterilise the Matrigel^®^-coated flask under UV exposure for 1 h.
6. Once set, use a sterile pipette tip to gently remove the top layer of liquid, ensuring not to disturb the Matrigel^®^ layer.

### 3.3. DPC Isolation

Note: For purposes of standardisation, extracted teeth should be free from caries or restorations. If the teeth are carious or have previously been restored, the pulp may have undergone physiological changes and the isolated DPCs may not be reliable for experimentation. Extracted teeth should be immediately stored in PBS for no more than 2 h.

**Figure 3:**
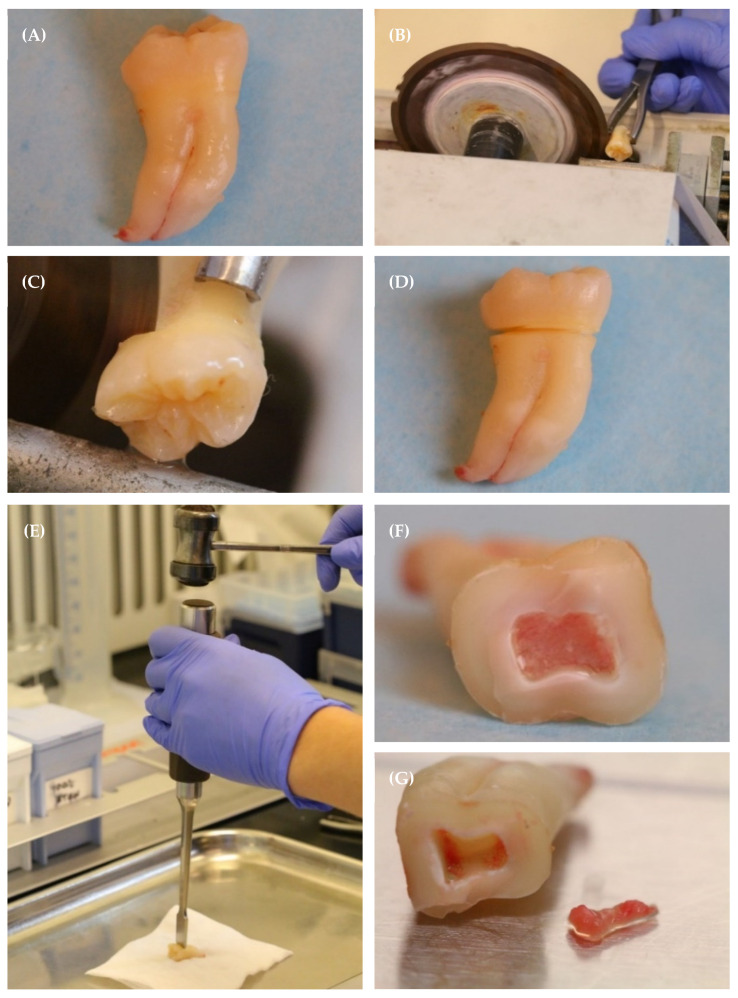
Extraction of dental pulp from the pulp chamber of a sound human third molar. (**A**) A sound third molar obtained following extraction from a healthy donor. (**B**–**D**) A groove is made around the CEJ using a water-cooled circular saw. (**E**,**F**) A surgical chisel is placed in the groove and tapped with a surgical mallet to separate the tooth along the CEJ and expose the pulp chamber. (**G**) The pulp is extirpated from the pulp chamber onto a sterile glass slide.

**Figure 4:**
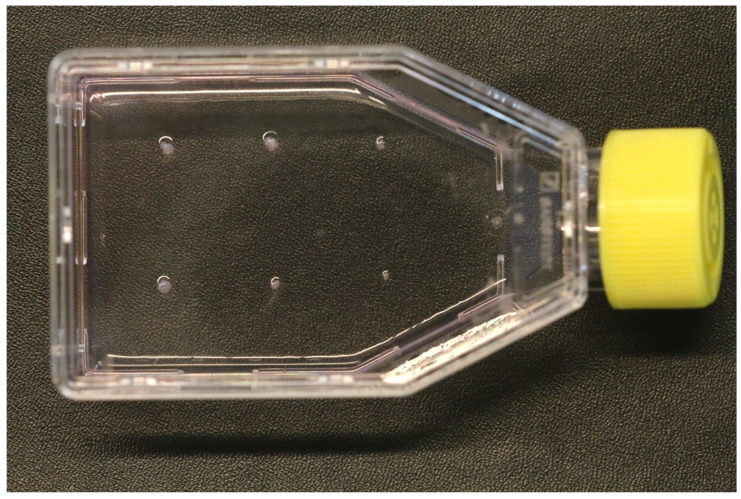
Six evenly spaced dental pulp tissue explants in a Matrigel^®^-coated T25 flask.

1. Transfer extracted sound third molars to 4ml PerioKin mouthwash in a sterile 50 mL centrifuge tube (Figure 3A) (See Note 2, Appendix A).
2. Incubate at room temperature for 5 min.
3. Transfer molars to 4 mL DMEM in a sterile 50 mL centrifuge tube for short-term storage during pulp extraction.
4. Using surgical forceps, position the tooth against the edge of the water-cooled saw, with the blade aligned with the cemento-enamel junction (CEJ). (See Notes 3 and 4, Appendix A) (Figure 3B,C).
5. Beginning with a low speed and light pressure, gently rotate the tooth to allow the saw to form a shallow groove around the circumference of the CEJ (Figure 3B,C).
6. Continue to rotate the tooth to allow the saw to deepen the groove until it is approximately 2 mm deep around the circumference of the CEJ (Figure 3D).
7. Place a sterile surgical chisel in the groove and gently tap with a sterile surgical mallet to separate the crown of the tooth from the root and expose the pulp chamber (Figure 3E,F).
8. Use a scalpel to gently remove the pulp from the pulp chamber onto a sterile glass slide (Figure 3G). Add 200 µL DMEM to ensure the pulp remains moist.

9. Use a sterile scalpel to slice the pulp into 5–6 small pieces, approximately 1 mm in size.
10. Use a scalpel to pick up the pulp explants and transfer them to the Matrigel^®^-coated flask at evenly spaced points (Figure 4). If required, a sterile scalpel may be used to make small grooves in the Matrigel^®^ layer to facilitate initial attachment, taking care not to extend the groove to the plastic base of the flask.
11. Incubate the flasks in a standard tissue culture incubator for 48 h, at which point 5 mL of cell culture medium can be added. The extended period of time following initial deposition of the explants into the flask until the first medium change is implemented in order to allow the explants to attach to the flask and to reduce the risk of dislodgement.
12. Change the medium initially after 7 days in culture. Thereafter, the medium may be changed every 3 days. Monitor the cells under phase contrast microscopy for explant outgrowth and signs of contamination or cell death. In the authors’ experience, cell outgrowth was typically observed after approximately 10 days in culture.
13. Once cellular outgrowth from the explants is established, the explants may be gently removed from the base of the flask with a sterile scalpel. Explants that do not display any signs of cell outgrowth may be discarded, while those that do display cellular outgrowth may be transferred to a sterile Matrigel^®^-coated T25 flask as outlined in steps 10–11. Explants can be reused in this manner until cell growth is no longer observed. In the authors’ experience, this typically occurred after the explant was reused three times.

### 3.4. Recovery of Cells from Matrigel^®^ for Further Subculture

When the cells reach 80% confluence, they should be isolated from the Matrigel^®^ coating as follows for subculture to allow further growth:

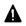
 **CRITICAL STEP** Use a sterile pipette to aspirate the cell culture medium from the flask, taking care not to disturb the cell monolayer or the Matrigel^®^ coating.
2. Add 2.5 mL pre-chilled Corning^®^ Cell Recovery solution to the flask (See Note 5, Appendix A.
3. Use a wide bore pipette tip to pipette the solution up and down and disrupt the Matrigel^®^ matrix.
4. Incubate at 4 °C for 20 min (See Note 6, Appendix A)
5. Aspirate Corning^®^ Cell Recovery solution, taking care not to aspirate Matrigel^®^ or disturb the cell monolayer.
6. Add 1 mL Trypsin-EDTA and gently rock the flask. Aspirate Trypsin-EDTA and add 1 ml fresh Trypsin-EDTA. Incubate the cells at 37 °C, 5% CO_2_ for 5–10 min prior to adding 1 mL of cell culture medium. A higher volume of cell culture medium may be used if preferred; however, in the authors’ experience, a 1:1 ratio of Trypsin-EDTA and cell culture medium is sufficient for recovery.
7. Transfer solution to a sterile 50 mL centrifuge tube and centrifuge at 100× *g* for 3 min.
8. Remove the supernatant and add 1 mL PBS. Centrifuge at 100× *g* for 3 min.
9. Repeat step 8.
10. Remove the supernatant and add 1 mL cell culture medium.
11. Transfer solution to a sterile T25 flask (passage 1). Incubate at 37 °C, 5% CO_2_ overnight before topping the medium up to 5 mL (See Note 7, Appendix A).

### 3.5. Subculture of Confluent Cells

When cells have reached 80% confluence in passage 1, they should be sub-cultured into T75 flasks as follows:

1. Aspirate the medium and add 1 mL Trypsin-EDTA. Gently rock the flask and aspirate Trypsin-EDTA prior to adding 1 mL fresh Typsin-EDTA. Incubate the cells at 37 °C, 5% CO_2_ for 5–10 min prior to adding 1 mL of cell culture medium.
2. Transfer the solution to a 50 mL centrifuge tube and centrifuge at 100× *g* for 3 min.
3. Discard the supernatant and resuspend the pellet in 2 mL culture medium. Aliquot the cell suspension into a sterile T75 flask (passage 2) and incubate overnight at 37 °C, 5% CO_2_. Top the medium up to 10 mL after 24 h (See Notes 7 and 8, Appendix A).

## 4. Expected Results and Discussion

The methods outlined here can be utilised to extract dental pulp from sound teeth for the expansion of DPCs using an explant culture method for in vitro experimentation or for further isolation of DPSCs. As part of this work, both an enzymatic digestion and an explant culture method were tested, and it was found that the explant culture method consistently produced sufficient cell numbers for further expansion and experimentation, while the enzymatic digestion method did not. Critically, the methods described here are designed to minimise physical and mechanical trauma to the pulp tissue. There is currently a lack of understanding surrounding the effects of mechanical stimulation on DPCs. While many studies have demonstrated a positive effect of mechanical stimulation on DPSC proliferation and differentiation, others have suggested that there is no effect, or indeed a negative effect on the biological properties of DPSCs [19]. Within the current study, efforts were made to minimize mechanical stimulation; however, there remains a lack of clarity regarding the response of DPCs to the various types of forces introduced in this study. A deeper understanding of these responses would allow for the further refinement and optimisation of this protocol, which would in turn allow for significant progress in the field of regenerative medicine.

**Figure 5:**
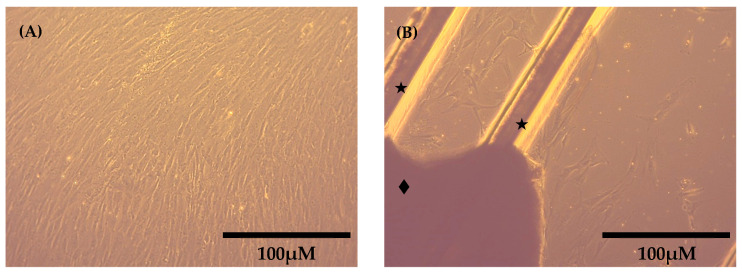
Cell growth in (**A**) a flask coated with 2mg/mL Matrigel^®^ and (**B**) an uncoated flask, with the explant (◆) and grooves made in the base of the flask with a scalpel to aid explant attachment (★) also visible.

In addition, a basement membrane is used to aid cell attachment and growth. The benefits of using Matrigel^®^ can be seen in Figure 5, which shows cell outgrowth in a Matrigel^®^-coated flask compared with an uncoated flask after 18 days in culture. Cell growth in the Matrigel^®^-coated flask is widespread, with neither the original explant nor the outer boundary of cell growth visible in the frame. In addition, the cells have reached confluence (Figure 4A). This is in contrast to the uncoated flask, in which a small number of isolated cells can be seen in close proximity to the explant (Figure 4B). 

Further isolation of DPSCs from DPCs is desirable for many areas of regenerative medicine. Initial attempts to isolate DPSCs used a colony picking method [1]; however, the colonies obtained were shown to be inconsistent in their expression of a number of markers, suggesting heterogeneity within the population. Currently, fluorescence-activated cell sorting (FACS) is widely accepted as a reliable method to isolate DPSCs. DPSCs can be identified based on their phenotype in relation to cell-surface antigens, such as the presence of CD29, CD44, CD73, and CD90, and absence or low expression of CD14, CD34 and CD45 [1,20]; however, it should be noted that many of these antigens are shared with other subsets of MSCs, and to-date DPSCs have not been demonstrated to express a unique immunophenotype.

The methods outlined here clearly and effectively describe the extraction of dental pulp from sound human third molars for cell culture and expansion. As the initial extraction and culture of DPCs was the primary aim of this paper, further characterisation of the cells isolated using these methods was not carried out. Therefore, future studies should focus on characterizing the cell population obtained using the isolation and culture methods described here, perhaps using FACS. In particular, the effect of the Matrigel^®^ layer on DPC proliferation and DPSC differentiation capacity should be investigated and quantified.

## Data Availability

No new data were created or analyzed in this study.

## Appendix A. Notes

1. A Matrigel^®^ concentration of 3 mg/mL is recommended by the supplier; however, in the current protocol, other concentrations were tested, including 2 mg/mL, which was found to be sufficient for explant attachment and growth. It should be noted that this concentration results in a fragile gel layer, and care should be taken at all times when handling the flask so as to not disturb the Matrigel^®^ coating.
2. Third molars are recommended as they are frequently extracted due to impaction or for orthodontic reasons and are therefore more likely to be free from caries or restorations than other teeth.
3. A water-cooled saw is necessary to minimise potential damage caused by high temperatures resulting from frictional heat and speed.
4. The CEJ is positioned more coronally on the mesial and distal aspect of a tooth. This anatomical demarcation curves apically on the buccal and lingual aspect of a tooth in a scalloped pattern. The cut should be positioned just beneath the most apical extent of the zenith of the scalloped cemento-enamel junction in order to de-roof the pulp chamber accurately.
5. There are a number of methods for depolymerisation of Matrigel^®^, including digestion with Corning^®^ Cell Recovery Solution or dispase; however, Corning^®^ Cell Recovery Solution is described here as it is recommended by the manufacturer where metabolism assays or RNA extraction are to be carried out.
6. The protocol described here is intended for use with primary human dental pulp cells isolated using an explant culture method. Different cells may require different incubation times with Corning^®^ Cell recovery solution.
7. Cells are initially seeded in a small quantity of culture medium to encourage settlement and enhance attachment to the base of the flask prior to topping medium up to final volume.
8. If required, DPSCs can be selected and isolated from the DPC population using FACS, as previously described [21].
